# Vascular endothelial growth factor facilitates the effects of telocytes on tumor cell proliferation and migration

**DOI:** 10.3389/fcell.2024.1474682

**Published:** 2024-11-13

**Authors:** Fujie Li, Xueying Tang, Haitao Cao, Wenya Wang, Chengyue Geng, Zuyao Sun, Xiaokun Shen, Shinan Li

**Affiliations:** ^1^ Liaoning Technology and Engineering Center for Tumor Immunology and Molecular Theranotics, Collaborative Innovation Center for Age-related Disease, Life Science Institute of Jinzhou Medical University, Jinzhou, China; ^2^ College of Basic Medical Science, Jinzhou Medical University, Jinzhou, China; ^3^ The First Affiliated Hospital of Jinzhou Medical University, Jinzhou, China; ^4^ College of Basic Medical Science, China Medical University, Shenyang, China

**Keywords:** telocytes, tumor growth, angiogenesis, vascular endothelial growth factor, bevacizumab

## Abstract

**Background:**

Telocytes, recently recognized as interstitial cells with a diverse range of potential functions, have attracted considerable attention for their involvement in tumorigenesis. Nevertheless, owing to certain challenges in the isolation and cultivation of telocytes, the research on telocytes has advanced rather slowly. Therefore, it is imperative to study the role and mechanisms of telocytes in tumors.

**Methods:**

We improved the separation method and successfully isolated telocytes by exploiting the combination of cell adhesion and magnetic bead sorting. Telocytes conditioned medium was collected to culture tumor cells and explore the role and mechanisms of telocytes in tumors.

**Results:**

MTT and colony formation assays demonstrated that telocytes promoted tumor cell proliferation. Wound healing experiments and transwell assays indicated that telocytes enhanced tumor cell migration. Real-time reverse transcriptase PCR analysis showed that the expression of E-cadherin was decreased, and that of Vimentin was notably increased. ELISA results revealed that telocytes secreted high levels of vascular endothelial growth factor (VEGF). And the promoting effects were alleviated by the VEGF inhibitor bevacizumab.

**Conclusion:**

Our findings revealed that telocytes promoted tumor cell proliferation, migration, and angiogenesis through VEGF. Notably, these effects were inhibited by the addition of bevacizumab. In conclusion, our findings illuminated the role of telocytes in promoting tumor progression, and confirmed their crucial regulatory role in the growth of tumor cells.

## Introduction

Telocytes (TCs) are a population of interstitial cells that were newly identified and officially named by the Popescu group in 2010 (Popescu and Faussone-Pellegrini, 2010). The study of TCs has made significant progress since the time of their discovery. Several functions of TCs have been demonstrated, including maintaining tissue homeostasis (Corradi et al., 2013; Rusu et al., 2012), tissue renewal and repair (Bei et al., 2015a), participating in intercellular communication (Gherghiceanu and Popescu, 2012), and immune regulation (Chen et al., 2013; Li et al., 2019), and still more functions remain to be explored. Using a variety of techniques, TCs have been found in various tissues and organs of different species, and they are usually present in the interstitial regions of different organs, such as the lung, heart, liver, and kidney (Kostin, 2016; Li et al., 2014; Ye et al., 2017; Zhao et al., 2016). Studies have found that TCs are involved in many pathologies, including cholelithiasis (Matyja et al., 2013), liver fibrosis (Fu et al., 2015), and psoriasis (Manole et al., 2015). In addition, TCs are closely related to tumors, such as breast cancer, basal cell carcinoma, squamous cell carcinoma, and extra-gastrointestinal stromal tumors (Mirancea et al., 2013; Mou et al., 2013; Varga et al., 2019). Although studies have revealed that TCs promoted the proliferation of breast cancer cells and inhibited the apoptosis of breast cancer cells (Mou et al., 2013; Smythies, 2015), their role is not fully understood in tumors.

The unique morphological feature of TCs that distinguishes them from “classical” stromal cells and endothelial cells is their extremely thin and long telopodes (Tps) with alternating thin segments (podomers) and dilated segments (podoms) (Popescu and Faussone-Pellegrini, 2010), which laid a good foundation for their multiple functions. TCs have been shown to contribute to a variety of functions in distinct tumors and activate different signaling pathways that have pivotal roles in physiological and pathological processes, especially in pathogenic evolution (Zhang and Xu, 2024). A mounting body of evidence suggests that TCs have an important role in tumorigenesis and undergo morphological changes or alterations in cell numbers under pathological conditions. For example, TCs hyperplasia has been reported in inflammatory fibroid polyp neoplasia and platelet-derived growth factor receptor α (PDGFRα) mutant gastrointestinal stromal tumors (Ricci et al., 2018), and the number of TCs was found to decrease in basal cell carcinoma and squamous cell carcinoma (Mirancea et al., 2013; Smythies, 2015). Although some reports have indicated specific effects of TCs on tumor growth, mechanisms underlying these effects are not fully elucidated. Therefore, it is imperative to study the role and mechanisms of TCs in tumors.

VEGF is a 40–45 kDa homodimeric protein. The VEGF family encompasses several members, such as VEGF-A, VEGF-B, VEGF-C, VEGF-D, VEGF-E, VEGF-F, placental growth factor (PLGF), and endocrine gland-derived VEGF (EG-VEGF) (Apte et al., 2019; Uemura et al., 2021). VEGF is secreted by a wide range of cells in both physiological and pathological circumstances. VEGF is predominantly secreted by tumor cells, certain stromal cells, and endothelial cells within the tumor microenvironment (TME) (Apte et al., 2019). The high expression of VEGF is associated with tumor recurrence, low survival rate, metastasis, and death (Eguchi and Wakabayashi, 2020; Jung et al., 2021). VEGF has been identified as a crucial factor governing endothelial cell sprouting, mitogenesis, cell migration, vasodilation, and vascular permeability (Patel et al., 2023). VEGF plays a significant role in vasculogenesis and it is essential for tumor growth and immunosuppression.

In this study, we used primary TCs to explore their effects on tumor cells and illustrated the possible underlying mechanisms. Our data demonstrated that TCs promoted tumor cell growth by enhancing cell proliferation, migration, and angiogenesis *in vitro* and *in vivo*, and VEGF produced by TCs mediated these processes.

## Materials and methods

### Animals

Six-week-old male C57BL/6 mice were used in the study. Mice were purchased from Beijing Vital River Laboratory Animal Technology Co., Ltd. and kept in the Jinzhou Medical University Animal Experiment Center. Mice were housed under specific pathogen-free conditions with a 12 h light/dark cycle and free access to food and water. Animal experiments and procedures were approved by the Animal Care and Use Committee of Jinzhou Medical University (no. 2020102001).

### Isolation and culture of TCs and preparation of conditioned medium (CM)

The hearts were removed under sterile conditions and placed in a 50 mL centrifuge tube with ice-cold complete medium. Then they were cut into 1 mm^3^ pieces and placed in a sterile culture dish. The tissue pieces were washed repeatedly with 1× phosphate buffered saline (PBS, Solarbio, cat. no. P1020) to remove the blood. An enzymatic digestion medium was prepared with 1 mg/mL collagenase type II (collagenase II, Sigma-Aldrich, cat. no. C6885), Dulbecco’s modified Eagle medium (DMEM)/F12 (Gibco, cat. no. C11330500BT), and 1% penicillin and streptomycin (PS, Solarbio, cat. no. P1400). The enzymatic digestion medium was added to the tissue, and the mixture was incubated at 37°C on a shaker at 120 rpm for 30 min. Then, 1× PBS was added to terminate the digestion and the mixture was centrifuged at 300 g for 10 min. The supernatant was discarded, and 1× PBS was added to resuspend the precipitate. The solution was filtered through a 70 μm nylon mesh (EMD Millipore) and centrifuged at 300 g for 10 min to isolate the cells. The cells were seeded onto 100 × 20 mm sterile culture dishes containing DMEM/F12, 10% fetal bovine serum (FBS, Gibco, cat. no. 10099141), and 1% PS and cultured at 37°C for 1.5 h to allow fibroblasts to attach to the dish (Li et al., 2016). The unattached cells (containing TCs) were purified by magnetic bead separation method according to the manufacturer’s instructions. TCs were seeded into sterile 60 × 15 mm culture dishes with DMEM/F12 complete medium and cultured in a cell incubator at 37°C with 5% CO_2_. TCs were observed daily by using an inverted microscope (Fresenius), and the medium was replaced every 3 days. When the density of TCs reached 80%, the medium was discarded and replaced with fresh complete medium, and the same amount of complete medium was added to a new 15 mL tube as the control group of TCs-CM. After culturing in the incubator for 72 h, the TCs-CM was collected, filtered, and centrifuged at 2,000 rpm for 5 min at 4°C for subsequent experiments. In this study, only isolated TCs at passages 0 or 1 were used for experiments.

### Cell lines and cell culture

Hepa1-6 cells were cultured with DMEM high glucose medium (Gibco, cat. no. C11995500BT), 10% FBS, and 1% PS. When the density of Hepa1-6 cells reached 80%, the medium was discarded and the cells were cultured with fresh DMEM complete medium for 72 h. The Hepa1-6-CM was collected, filtered, and centrifuged at 2,000 rpm for 5 min at 4°C for subsequent experiments. B16-F10 cells were cultured with DMEM high glucose medium, 10% FBS, and 1% PS. RM-1 cells were cultured with RPMI 1640 medium (Gibco, cat. no. C11875500BT), 10% FBS, and 1% PS. SVEC4-10 cells were purchased from BeNa Culture Collection and cultured with DMEM high glucose medium, 10% FBS, and 1% PS. All cells were cultured at 37°C in a 5% CO_2_ cell incubator.

### Immunofluorescence staining

Immunofluorescence staining was carried out as described (Jiang et al., 2018). The primary antibodies (Armenian Hamster anti-CD34, rat anti-CD140a) were purchased from BioLegend (cat. no. 119307 and cat. no. 135908). The secondary antibodies (DyLight™ 594 goat anti-hamster IgG, FITC-conjugated goat anti-rat IgG) were purchased from BioLegend and ZSGB-Bio, respectively (cat. no. 405504 and cat. no. ZF-0311).

### MTT assay

The cell viability was assessed by MTT assay. Briefly, RM-1 cells, Hepa 1-6 cells, and B16-F10 cells were seeded in 96-well plates at a density of 1 × 10^3^ cells per well. After the adhesion of cells, replaced the medium with TCs-CM and cultured at 37°C in a 5% CO_2_ cell incubator. Following incubation, 20 µL MTT solution (Solarbio, 5 mg/mL, cat. no. M8180) was added to each well and incubated for 4 h at 37°C at each time point, including 48 h, 72 h, 96 h, and 120 h. Discarded the liquid in the wells, and 150 µL DMSO (Santa Cruz Biotechnology, cat. no. sc-358801) was added to dissolve the formazan crystals. And measured by an automated microplate reader (Thermo Scientific) at 450 nm.

### Apoptosis assay

Cells were collected, washed with 1 × PBS, stained with the Annexin V-FITC Apoptosis Kit (BioLegend, cat. no. 640922), and detected by flow cytometry. Briefly, 1 × 10^5^ Hepa 1-6 cells were seeded in 24-well plates and cultured with TCs-CM for 72 h. Hepa 1-6 cells were digested with 0.25% Trypsin-EDTA (Gibco, cat. no. 25200072) and collected. The Annexin V-FITC Apoptosis Kit was used to stain cells for 15 min at room temperature in the dark. The percentage of cell apoptosis was analyzed by a FACSVerse flow cytometer.

### Colony formation assay

Hepa 1-6 cells were seeded in 6-well plates. After the adhesion of Hepa 1-6 cells, replaced the medium with TCs-CM. Placed in a cell incubator and cultured for 10 days until colonies form. The plate was washed with 1 × PBS. 4% paraformaldehyde was used to fix cells for 30 min. Next, they were dyed with 0.1% crystal violet for 15 min at room temperature. The colonies were observed with an inverted microscope and counted using ImageJ software.

### ELISA

TCs-CM and Hepa 1-6-CM were collected as described above. The relative expression levels of Interleukin- 6 (IL-6), VEGF, macrophage inflammatory protein-2 (MIP-2), monocyte chemoattractant protein-1 (MCP-1), and matrix metalloproteinase 9 (MMP-9) in CM were analyzed by ELISA kits (Shanghai Enzyme-linked Biotechnology Co., Ltd.), and the absorbance values were detected at 450 nm wavelength. All operations were performed according to the manufacturer’s instructions.

### Wound healing assay

The Hepa 1-6 cells (5 × 10^5^ cells/well) were seeded in 12-well plates and incubated overnight to reach a fusion rate of 100%. After Hepa 1-6 cells had grown to 100% confluence, the monolayers were scratched with a 200 µL pipette tip, and the cell debris was removed by washing with 1×PBS, and cultured with TCs-CM. Cell images were captured at 0 h, 24 h, 48 h, and 72 h, respectively, and ImageJ software was used to evaluate the area of cell scratches.

### Transwell assay

Hepa 1-6 cells (5 × 10^4^ in 200 μL serum-free medium), were seeded into the upper chamber of a transwell insert (8 μm pore size; Corning Inc., cat. no. 3422), and the TCs-CM was added in the lower chamber (600 μL/well) as indicated. After incubation at 37°C, 5% CO_2_ for 24 h, the transwell chamber was taken out and the medium in the well was discarded and washed with 1 × PBS. Subsequently, the cells were fixed with 4% paraformaldehyde for 30 min and stained with 0.1% crystal violet for 30 min. The upper unmigrated cells were gently wiped off with a cotton swab, and counted under the microscope. Five fields of view were randomly selected to calculate the number of migrated Hepa 1-6 cells.

### Real-time reverse transcriptase PCR analysis

Hepa 1-6 cells were seeded in 12-well plates at a density of 5 × 10^4^ cells per well and cultured with TCs-CM for 72 h. RNA was extracted using Trizol reagent (Invitrogen, cat. no. 15596018CN) according to the manufacturer’s instructions and quantified using a Nanodrop 1,000 (Thermo Fisher). RNA was converted to cDNA by using a PrimeScript ™ RT reagent kit (Takara, cat. no. RR037A) according to the manufacturer’s instructions. Real-time reverse transcriptase PCR analysis was performed in a 20 μL reaction volume with TB Green Premix Ex Taq™ (Takara, cat. no. RR420A) according to the manufacturer’s protocols. The *GAPDH* gene was used as a reference. Relative expression of *Cdh1* and *Vim* was calculated using the 2^−ΔΔCT^ method. The primer sequences are listed in Table 1.

**TABLE 1:** Sequence of mouse-derived primers.

Gene name	Sequence of primer (5′–3′)
*Gapdh*	F:ATTGTCAGCAATGCATCCTG
	R:ATGGACTGTGGTCATGAGCC
*Vim*	F:TCAAGACTCGGTGGACTTCT
	R:CGCACCTTGTCGATGTAGTT
*Cdh1*	F:CTGCTGCTCCTACTGTTTCTAC
	R:TCTTCTTCTCCACCTCCTTCT

### Bevacizumab blocking experiment

Bevacizumab (TargetMol Chemicals Inc., cat. no. T9904, 200 μg/mL) was added to the above colony formation assay, wound healing assay, and transwell assay, and other procedures remained unchanged.

### Angiogenesis experiment

Matrigel (Solarbio, cat. no. M8370) was diluted with pre-cooled serum-free medium, added to 96-well plates (50 μL/well), and incubated in the cell incubator for 30 min. Subsequently, 2 × 10^4^ suspended SVEC4–10 cells were seeded on the top of matrigel and incubated at 37°C for 6 h. The angiogenesis experiment results were captured using a microscope and analyzed with ImageJ software.

### Tumor model constructed *in vivo*

Hepa 1-6 cells were suspended with medium and then injected into the livers of six-week-old male C57BL/6 mice to establish a hepatocellular carcinoma model (1 × 10^6^ cells/mouse). Simultaneously, 200 μL medium containing 2.5 × 10^5^ TCs or 200 μL 1 × PBS was injected into the tail veins of mice weekly for a total of two times. The mice were euthanized on day 28 after the first injection, and their livers were resected, weighed, photographed, and subjected to statistical analysis.

### Statistical analysis

All statistical analyses were performed using the Student’s *t*-test and Two-way ANOVA analysis according to the design of experiment. The GraphPad Prism 8.0 software was used to perform statistical analysis. The *P* values of <0.05 were considered significant (*P < 0.05, **P < 0.01, ***P < 0.001, and ****P < 0.0001.

## Results

### Separation and purification of TCs by magnetic bead sorting

**FIGURE 1:**
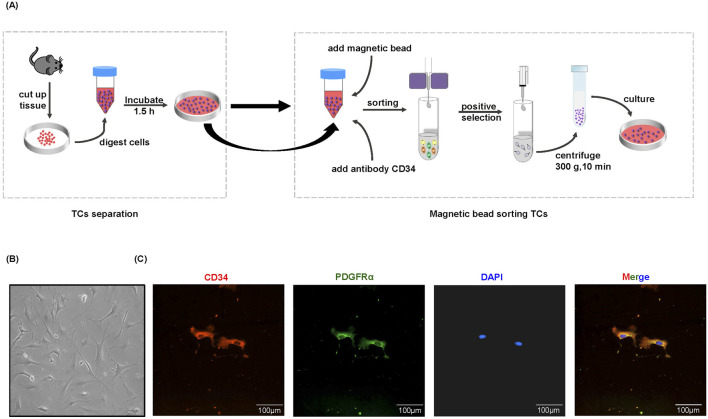
Separation and immunofluorescence staining of TCs. **(A)** Flow chart of magnetic bead sorting TCs. **(B)** The representative morphology of TCs under a light microscope. The images were acquired 96 h after purification and culture. **(C)** Immunofluorescence staining for CD34 (red) and PDGFRα (green) with DAPI for nuclear.

To gain valuable insight into the potential functions of TCs, it is evident that *in vitro* analyses are necessary. However, there is currently a paucity of effective techniques for the isolation and purification of TCs, and the protocols for TCs isolation are still in nascent stages and lack full standardization (Li et al., 2016; Jiang et al., 2018; Bei et al., 2015b; Cretoiu et al., 2015; Hatta et al., 2012; Song et al., 2019; Yang et al., 2017; Zheng et al., 2013; Romano et al., 2020). In this study, we developed a methodology to isolate and purify TCs *in vitro*. In brief, we isolated TCs from mice using a combination of cell adhesion properties and magnetic bead sorting (Figure 1A). TCs were identified by their morphology after primary culture (Figure 1B), based on their small cell body (Tc) and extremely long and thin Tps. Although transmission electron microscopy is generally considered to be the gold standard method, double immunolabeling of TCs is important for distinguishing them from other types of interstitial cells (Fu et al., 2015; Cantarero et al., 2016). CD34/PDGFRα double positive immunostaining has been demonstrated to be a marker for cardiac TCs (Zhou et al., 2015). The results showed that isolated TCs were double positive for CD34 and PDGFRα, and the morphological structure of TCs was identifiable (Figure 1C).

### TCs promoted the proliferation of tumor cells

**FIGURE 2:**
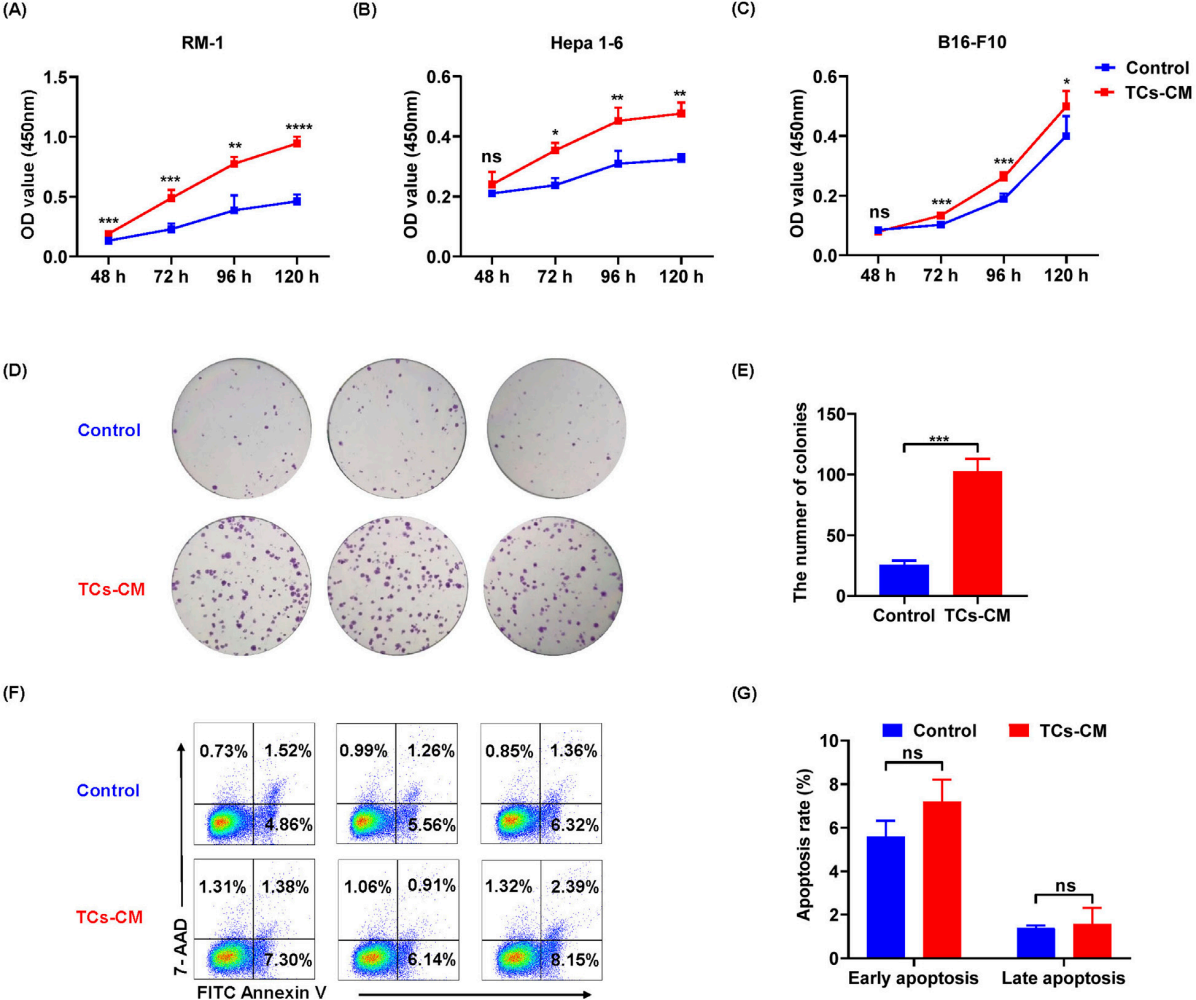
The influence of TCs on the proliferation and apoptosis of tumor cells. **(A–C)** The viability of RM-1 cells, Hepa 1-6 cells, and B16-F10 cells was assessed at various time points after exposure to TCs-CM. (The control group was cultured in normal complete medium, and it remained the same in the subsequent experiments). **(D)** Hepa 1-6 cells were seeded in 6 well-plates at a density of 500 cells per well and cultured with TCs-CM for 10 days to assess colony formation. **(E)** Quantitative analysis of the number of Hepa 1-6 cell colonies was conducted. **(F)** Hepa 1-6 cells were cultured with TCs-CM for 72 h, and cell apoptosis was measured by flow cytometry. **(G)** The apoptosis proportion of Hepa 1-6 cells in different groups was assessed. Data were represented as the mean ± SEM. ns, not significant; *P < 0.05, **P < 0.01, ***P < 0.001, ****P < 0.0001.

We wanted to understand how TCs affected tumor cell progression. We collected TCs-CM for culturing different tumor cells (including RM-1 cells, Hepa 1-6 cells, and B16-F10 cells). The results of MTT assay showed that TCs-CM could significantly promoted the proliferation of different tumor cells compared with the control group (Figures 2A–C), indicating that the tumor-promoting effect of TCs was nonspecific. Consequently, we next used Hepa 1-6 cells to explore the impact of TCs on tumor cells. We analyzed the capacity of Hepa1-6 cells to form colonies when cultured with or without TCs-CM. Compared with the control group, the number of colonies in the TCs-CM group was clearly increased (Figures 2D, E), and the percentage of cell apoptosis between the two groups appeared to have no difference (Figures 2F, G). These results demonstrated that TCs have a significant role in regulating the proliferation of Hepa 1-6 cells, thereby facilitating tumor cell growth.

### TCs promoted the migration of tumor cells

**FIGURE 3:**
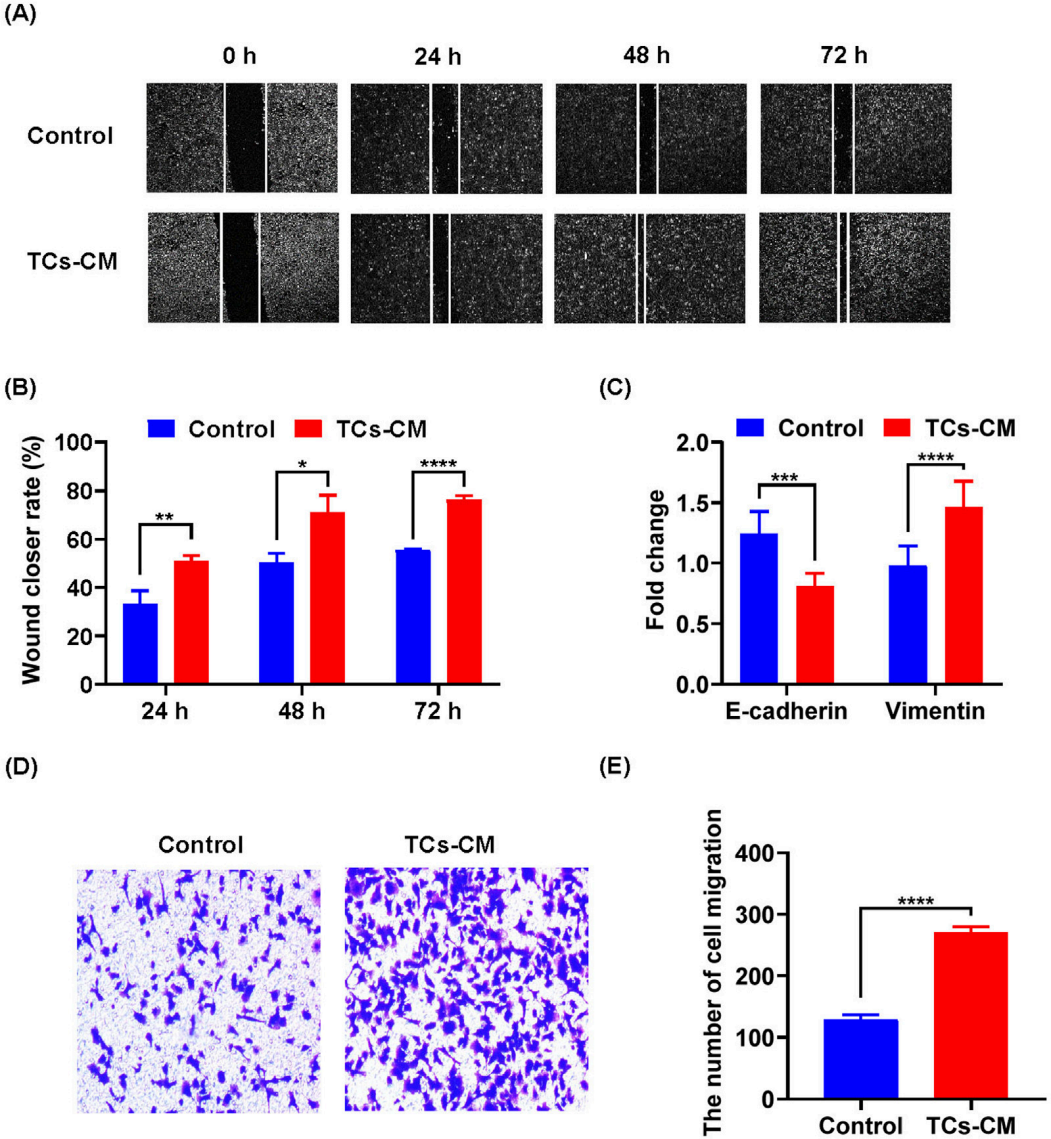
The effect of TCs on the migration of tumor cells. **(A)** Representative images of Hepa 1-6 cells cultured with TCs-CM were captured at 0 h, 24 h, 48 h, and 72 h after creating a scratch on the cell surface (×4). **(B)** Statistical analysis was performed on the scratch width. **(C)** Hepa 1-6 cells were cultured with TCs-CM for 72 h, and real-time reverse transcriptase PCR analyses were conducted to measure the gene expression levels of *Cdh1* and *Vim*. **(D)** Migrated Hepa 1-6 cells that were cultured with TCs-CM for 24 h in the Transwell assay were shown in representative images (×10). **(E)** Statistical analysis was performed on the migrated Hepa 1-6 cells. Data were represented as the mean ± SEM. *P < 0.05, **P < 0.01, ***P < 0.001, ****P < 0.0001.

Based on these findings, we demonstrated that TCs modulated the proliferation of tumor cells. Next, we evaluated the effect of TCs on tumor cell migration. According to the results of wound healing assay, the Hepa 1-6 cell migration was enhanced in the TCs-CM group (Figures 3A, B). Loss of *Cdh1* (E-cadherin) expression is considered a key event in EMT (Puisieux et al., 2014). Real-time reverse transcriptase PCR analysis showed that the expression *of* *Cdh1* was decreased, and that of *Vim* (vimentin) was notably increased (Figure 3C), which indicated that Hepa 1-6 cells had stronger migration ability in TCs-CM group. Meanwhile, the transwell assay revealed increased number of migrated Hepa 1-6 cells in the TCs-CM group (Figures 3D, E). These results confirmed that TCs significantly promoted Hepa 1-6 cell migration.

### TCs increased VEGF in TCs-CM

**FIGURE 4:**
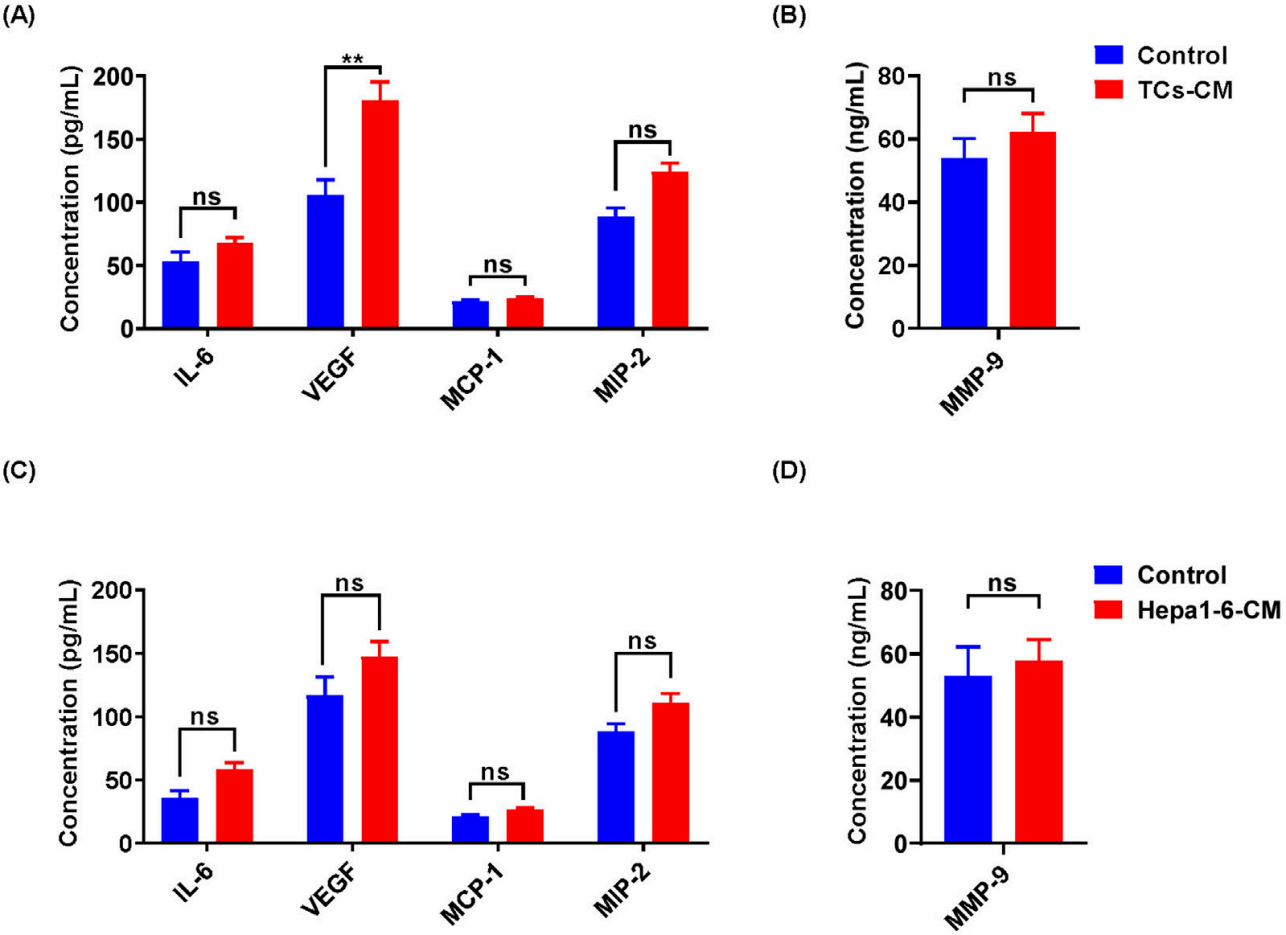
The cytokine expression in TCs-CM and Hepa 1-6-CM was detected by ELISA. **(A, B)** The expression levels of IL-6, VEGF, MCP-1, MIP-2, and MMP-9 in TCs-CM were assessed. When the density of TCs reached 80%, the medium was discarded and cultured with fresh complete medium for 72 h. Collected TCs-CM and detected. **(C, D)** Similarly, when the density of Hepa 1-6 cells reached 80%, they underwent the same processes to collect and analyze Hepa 1-6-CM. Data were represented as the mean ± SEM. ns, not significant; **P < 0.01.

These results indicated that TCs significantly promoted the proliferation and migration of Hepa 1-6 cells. Studies have revealed that TCs could secrete numerous factors, including cytokines, chemokines, and extracellular vesicles, to enhance their various functions (Albulescu et al., 2015). We were curious about whether the promotional effect we observed was associated with cytokines secreted by TCs. For this purpose, IL-6, VEGF, MCP-1, MIP-2, and MMP-9 levels were examined in TCs-CM and Hepa 1-6-CM. We observed a significant increase in the expression of VEGF in the TCs-CM group compared with the control group. However, there was no discernible change in the expression of VEGF in the Hepa 1-6-CM group (Figures 4A–D), indicating that the upregulation of VEGF was TCs-specific. In addition, there was no significant difference in the expression of IL-6, MCP-1, MIP-2, and MMP-9 in the TCs-CM group or the Hepa 1-6-CM group. The results suggested that TCs secreted high levels of VEGF, which may promote tumor progression.

### Bevacizumab inhibited the promoting effect of TCs on tumor cell proliferation, migration, and angiogenesis

**FIGURE 5:**
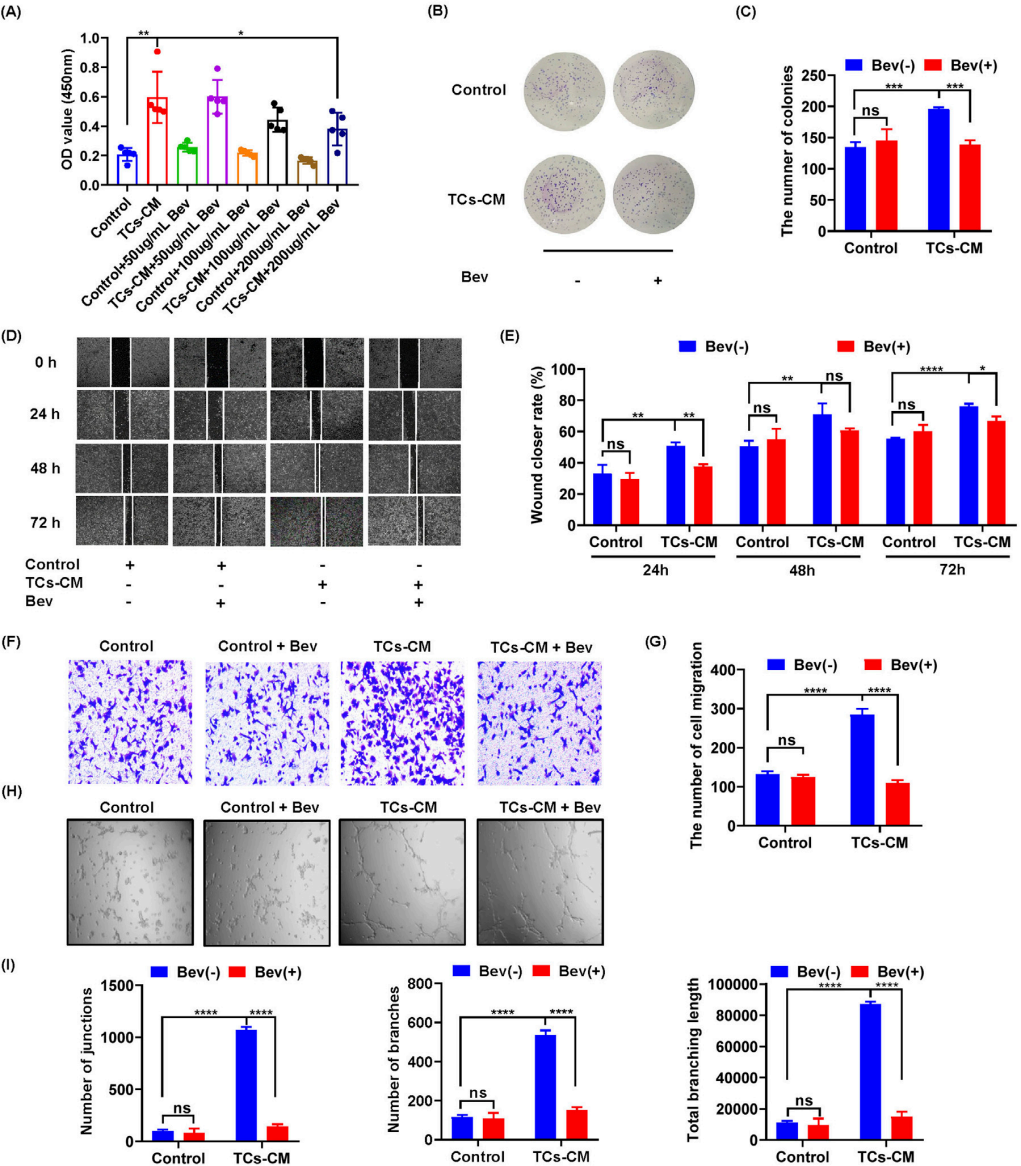
The effect of bevacizumab on the promotion of TCs in Hepa 1-6 cells. **(A)** The viability of Hepa 1-6 cells was assessed using the MTT assay following treatment with bevacizumab at concentrations of 0, 50, 100, and 200 μg/mL for a duration of 72 h. **(B)** Hepa 1-6 cells were seeded in 6-well plates at a density of 1,000 cells per well with or without bevacizumab and cultured for 10 days to observe colony formation. **(C)** Quantitative analysis was performed to determine the number of Hepa 1-6 cell colonies. **(D)** Hepa 1-6 cells were cultured with TCs-CM in the presence or absence of bevacizumab, and representative images were captured by microscopy at time points of 0 h, 24 h, 48 h, and 72 h after creating scratches on the cell surface (×4). **(E)** Statistical analysis was conducted on the scratch width. **(F)** Migrated Hepa 1-6 cells that were cultured with TCs-CM for 24 h with or without bevacizumab were presented in representative images (×10). **(G)** Statistical analysis was performed on the migrated Hepa 1-6 cells. **(H)** Angiogenesis experiments were carried out using SVEC4-10 cells cultured with TCs-CM and incubated for 6 h with or without bevacizumab (×4). **(I)** Quantitative analyses were performed to assess the number of junctions, branches, and total branching length. Data were represented as the mean ± SEM. ns, not significant. *P < 0.05, **P < 0.01, ***P < 0.001, ****P < 0.0001.

The combination of VEGF and VEGFR initiates various intracellular signaling pathways, regulating vascular permeability and endothelial cell survival, proliferation, and migration, thereby promoting tumor cell growth and accelerating tumor progression (Apte et al., 2019). The high expression of VEGF in the TCs-CM group captured our attention, prompting us to hypothesize that TCs may contribute to the acceleration of tumor progression through VEGF. To verify our hypothesis, we used bevacizumab to investigate the mechanism by which TCs regulate the proliferation and migration of tumor cells. The results showed that the promotion of Hepa 1–6 cell proliferation increased in the presence of TCs but was inhibited after adding bevacizumab (Figures 5A–C). However, there was no significant change observed in the proliferation of Hepa 1-6 cells in the control group. These findings indicated that bevacizumab mitigated the stimulatory influence of TCs on the proliferation of Hepa 1-6 cells. We further investigated the effect of bevacizumab on the migration of Hepa 1-6 cells by the wound healing assay and the transwell assay. The results showed that the increased migration of Hepa 1-6 cells induced by TCs was suppressed by bevacizumab (Figures 5D–G). Similarly, there was no discernible difference in the migration ability of Hepa 1-6 cells in the control group, suggesting that bevacizumab inhibited the migration-promoting effect of TCs on Hepa 1-6 cells. Our results demonstrated that TCs significantly enhanced both proliferation and migration of Hepa 1-6 cells, and that effect was mitigated by bevacizumab.

We further elucidated how TCs were involved in angiogenesis—a crucial process for tumorigenesis. The results revealed a more pronounced angiogenic response when SVEC 4–10 cells were exposed to TCs-CM compared with those in the control group. Furthermore, the addition of bevacizumab completely attenuated TCs-promoted angiogenesis in SVEC4–10 cells (Figures 5H, I). These results suggested that bevacizumab attenuated processes such as proliferation, migration, and angiogenesis promoted by TCs.

### TCs promoted the growth and progression of tumors *in vivo*

**FIGURE 6:**
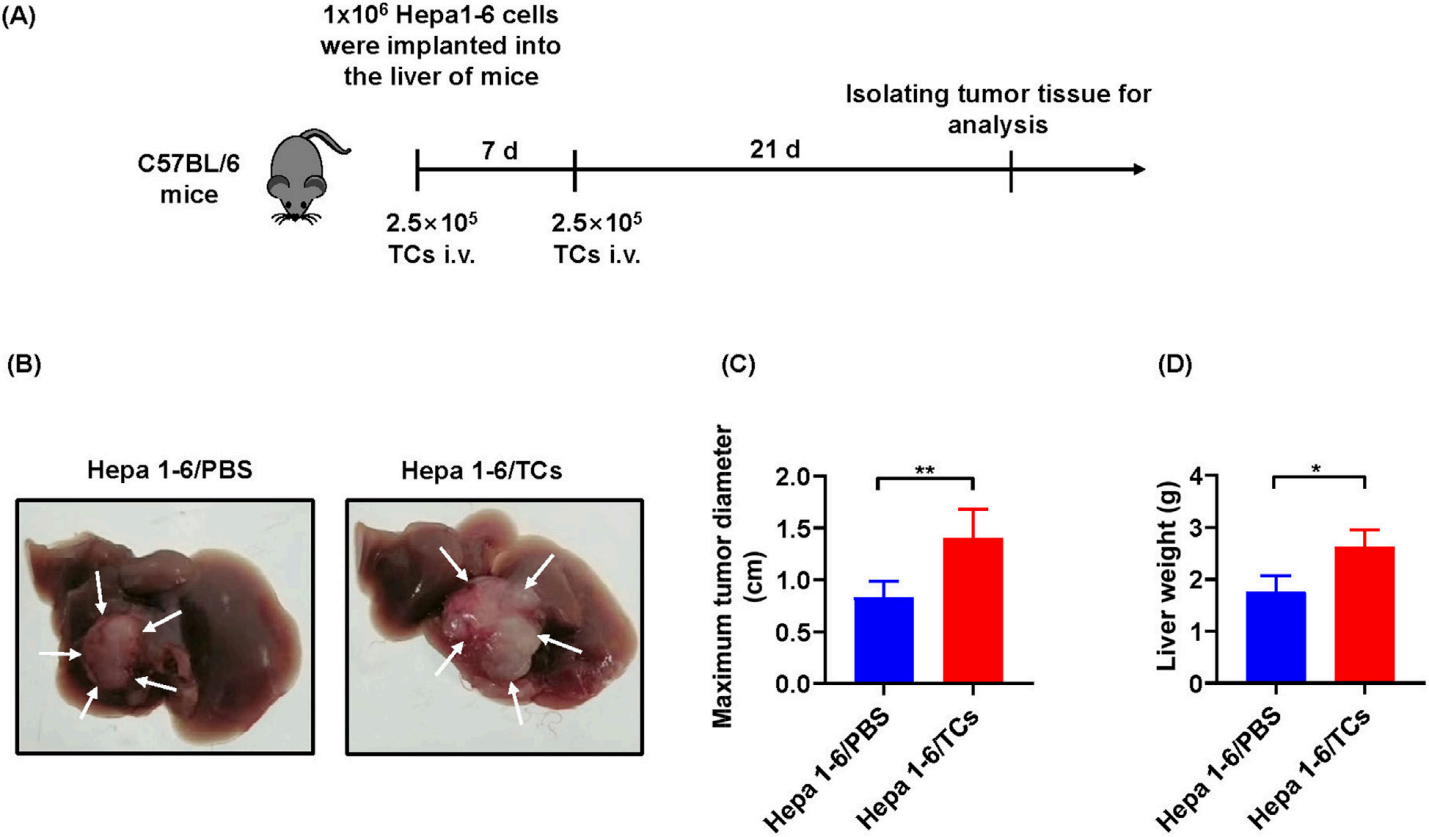
The effects of TCs on tumor growth *in vivo*. **(A)** The schematic diagram illustrated the construction processes of hepatocellular carcinoma tumor model in C57BL/6 mice. **(B)** The images depicted the harvested tumor from one representative experiment at 28 days. **(C)** Statistical analysis was conducted on the maximum tumor diameter of hepatocellular carcinoma in mice from different groups, measured using a caliper. **(D)** Statistical analysis was performed on the liver weight of hepatocellular carcinoma in mice from different groups. Data were represented as the mean ± SEM. *P < 0.05, **P < 0.01.

As the *in vitro* findings in this study demonstrated that TCs significantly promoted the growth of tumor cells, we next investigated their effects *in vivo*. We further established a mouse model of hepatocellular carcinoma by implanting Hepa1-6 cells *in situ*. At 28 days post implantation, a successful mouse model of hepatocellular carcinoma was established (Figure 6A). Compared with mice treated with 1×PBS, those treated with TCs exhibited a markedly increased in maximum tumor diameter (Figures 6B, C) and liver weight (Figure 6D), indicating that TCs accelerated the growth and progression of the tumors.

## Discussion

In this study, we presented a novel mechanism by which TCs facilitate tumor development. Our data disclosed that TCs notably promoted the proliferation and migration of tumor cells, leading to an increase in the size of the tumor and its progression. The accelerated growth of tumor cells induced by TCs was associated with increased VEGF secretion. Furthermore, these effects were alleviated by bevacizumab.

TCs are a population of interstitial cells that were newly identified and officially denominated by the Popescu group in 2010 Popescu and Faussone-Pellegrini (2010). Since the discovery of TCs, certain challenges have emerged in their isolation, purification, and culture of TCs. To date, diverse techniques for isolating TCs have been proposed, including enzymatic digestion, flow cytometric sorting, and magnetic bead sorting (Li et al., 2016; Romano et al., 2020; Albulescu et al., 2015). Flow cytometry sorting requires a considerable number of cells and impacts cell viability. By contrast, the magnetic bead sorting method is relatively efficient and straightforward. Therefore, building on these original experiments, our study enhanced the experimental approaches for separating and purifying TCs. Our method effectively isolated TCs while preserving cell viability. TCs can interact with parenchyma cells, immune cells, and tumor cells via their Tps. Furthermore, TCs might be accountable for the origin of certain malignancies (Ratajczak et al., 2016). In cancer specimens, TCs exhibit gene heterogeneity on specific chromosomes, indicating their diverse impacts on tumor cell behaviors, such as cell signaling, proliferation, movement, tumorigenesis, and inflammatory resistance (Luan and Xu, 2023; Zhu et al., 2015). Studies regarding the morphological and functional changes of TCs in the gastrointestinal stromal tumor clarified that TCs were the physiological counterpart of both inflammatory fibroid polyp and PDGFRA‐mutant gastrointestinal stromal tumor, possibly pathogenetically associated with both of these tumor types (Ricci et al., 2018). It was discovered that TCs facilitated hepatocellular carcinoma metastasis, and the activation of the Raf/ERK signaling pathway and the downregulation of mi942-3p played crucial roles in enhancing MMP9 expression and promoting hepatocellular carcinoma metastasis, respectively (Xu et al., 2021). In our research, we confirmed a promoting effect of primary TCs on tumor cell growth. The MTT and colony formation assays in this study demonstrated that TCs promoted tumor cell proliferation. In addition, wound healing experiments and transwell assays indicated that TCs enhanced tumor cell migration. And the expression levels of *Cdh1* and *Vim* in real-time reverse transcriptase PCR analysis further confirmed these findings. Overall, our study systematically clarified the promoting effect of primary TCs on tumor cells. Moreover, an ELISA analysis revealed a high expression of VEGF in the TCs-CM group, which attracted our attention.

VEGF has a significant role in tumor angiogenesis and influences tumor progression. VEGF and its receptors (VEGFRs) play a crucial role in the development of the vascular system and the maintenance of vascular integrity. When VEGF is overexpressed, it may also contribute to related pathological processes, such as unrestricted angiogenesis and cellular metastasis in numerous types of cancer (Peng et al., 2019). Evidence suggests that VEGF operates in tumors not only by promoting angiogenesis but also by directly impacting cancer cells (Frezzetti et al., 2017). Despite the evidence from studies indicate that VEGF is overexpressed in the majority of solid tumors, such as colorectal cancer (Zhao et al., 2018) and oral cancer (Yanase et al., 2014), the cell origin of VEGF has not been fully investigated. In our study, the expression of VEGF in Hepa 1-6- CM and TCs-CM was detected by ELISA. The ELISA results revealed an increased in VEGF levels in the TCs-CM group, while there was no significant change in the control group. Previous studies have reported higher expression of IL-6 and VEGF in mouse cardiac TCs-CM compared with 3T3 fibroblasts, suggesting a potential regulatory role of TCs in cell growth, myocyte differentiation, and angiogenesis (Albulescu et al., 2015). Based on its biological functions, we hypothesized that the stimulatory effects of TCs on tumor cell growth and migration may be attributed to an upregulation of VEGF secretion. To verify this hypothesis, we used bevacizumab in our study. Our findings showed that the stimulatory effect of TCs on tumor cell growth and migration was attenuated upon the addition of bevacizumab. However, there was no significant change observed in the control group. These results suggested that VEGF produced by TCs significantly promoted the proliferation and migration of tumor cells. Furthermore, studies demonstrated that the number of TCs were significantly increased around the newly formed blood capillaries during embryonic lung development and their Tps connected with endothelial cells, which provided clear evidence regarding the role of TCs in pulmonary angiogenesis, formation of the air-blood barrier, organization, and development of many lung structures in prenatal period of lung development (Hussein and Mokhtar, 2018; Zheng and Wang, 2016). Another study illustrated the involvement of TCs in angiogenesis during quail embryonic development (Soliman, 2021).

Angiogenesis, the intricate process of creating new blood vessels in the existing vascular system, is tightly regulated by numerous pro-angiogenic and anti-angiogenic factors under normal physiological conditions (Lin et al., 2016). Atypical cells can produce elevated levels of pro-angiogenic factors, leading to the formation of abnormal vascular networks in immature blood vessels, producing a hypoxic microenvironment (Viallard and Larrivee, 2017). These factors increase the risk of aggressive tumor cell formation and weaken the immune response. The occurrence, progression, and metastasis of tumors were shown to be accompanied by excessive expression of pro-angiogenic factors that played crucial roles in local angiogenesis (Aleksandrovych and Gil, 2021). Our angiogenesis experiments demonstrated that TCs facilitated vascular angiogenesis, thereby expediting tumor progression, and this effect was suppressed by bevacizumab.

In summary, our investigation has revealed that TCs promote tumor cell proliferation, migration, and angiogenesis by upregulating VEGF secretion, providing new insights into the role of TCs in tumorigenesis.

## Data Availability

The original contributions presented in the study are included in the article/supplementary material, further inquiries can be directed to the corresponding authors.

## Abbreviations

TCs, telocytes; CM, conditioned medium; TCs-CM, telocytes conditioned medium; Tps, telopodes; PDGFRα, platelet-derived growth factor receptorα; VEGF, vascular endothelial growth factor; PLGF, placenta growth factor; EG-VEGF, endocrine gland-derived VEGF; TME, tumor microenvironment; IL-6, interleukin 6; MIP-2, macrophage inflammatory protein-2; MCP-1, monocyte chemoattractant protein-1; MMP9, matrix metallopeptidase 9.
